# Assessment of cognitive function in individuals with Down syndrome and dementia: a systematic review

**DOI:** 10.1590/1980-5764-DN-2025-0308

**Published:** 2026-02-13

**Authors:** Ana Clara Lira do Nascimento, Egeval Pereira da Paz, Graziela Rosa Lopes Bastos Freire, Sonja Costa Coelho Gayoso e Almendra, Maria Costa Coelho Gayoso e Almendra, Lívia Fabrícia Francisco de Lima, Ludmila Belo Ramos da Silva, Júlia Feitosa Brito dos Santos, Bianca Alves de Paiva, Luís Eduardo Soares Sampaio Novaes, Matheus Mendes de Melo, Ana Iza Gomes da Penha Sobral

**Affiliations:** 1 Universidade de Pernambuco, School of Medicine, Group of Geriatrics, Recife PE, Brazil.; 2 Universidade Estadual do Piaui, School of Medicine, Teresina PI, Brazil.; 3 Universidade de Pernambuco, Department of Occupational Therapy, Recife PE, Brazil.

**Keywords:** Down Syndrome, Cognition, Alzheimer Disease, Dementia, Systematic Review, Síndrome de Down, Cognição, Doença de Alzheimer, Demência, Revisão Sistemática

## Abstract

Individuals with Down syndrome (DS) face a high risk of dementia, particularly Alzheimer’s disease (AD). Early diagnosis is complex due to pre-existing cognitive deficits, underscoring the need for specific diagnostic criteria.

**Objective:**

To present cognitive and behavioral features of dementia in DS and examine cognitive assessment tools.

**Methods:**

Following Preferred Reporting Items for Systematic Reviews and Meta-Analyses (PRISMA) guidelines, the databases United States National Library of Medicine (PubMed), Scopus, Web of Science, and Virtual Health Library (BVS) were searched for studies (2014-2024) in English, Portuguese, or Spanish. The quality of the included articles was assessed using the Strengthening the Reporting of Observational Studies in Epidemiology (STROBE) criteria.

**Results:**

Of 254 records screened, 28 studies met the inclusion criteria. Early dementia in DS presents with declines in executive functions, memory, language, attention, and behavioral changes. Behavioral symptoms, including agitation, hallucinations, and apathy, were prominent in AD-DS and prodromal dementia, and may serve as early indicators.

**Conclusion:**

Early dementia in individuals with DS is characterized by cognitive and behavioral declines, with behavioral symptoms potentially serving as early diagnostic markers.

## RESUMO.

Indivíduos com síndrome de Down (SD) apresentam alto risco de demência, particularmente a doença de Alzheimer (DA). O diagnóstico precoce é complexo em razão dos déficits cognitivos preexistentes, destacando a necessidade de critérios diagnósticos específicos.

**Objetivo:**

Apresentar características cognitivas e comportamentais da demência na SD e analisar instrumentos de avaliação cognitiva.

**Métodos:**

Seguindo as diretrizes Preferred Reporting Items for Systematic Reviews and Meta-Analyses (PRISMA), as bases de dados United States National Library of Medicine (PubMed), Scopus, Web of Science e Biblioteca Virtual em Saúde (BVS) foram pesquisados estudos (2014-2024) em inglês, português ou espanhol. A qualidade dos artigos incluídos foi avaliada utilizando os critérios Strengthening the Reporting of Observational Studies in Epidemiology (STROBE).

**Resultados:**

Dos 254 registros analisados, 28 estudos atenderam aos critérios de inclusão. A demência precoce na SD apresenta declínios em funções executivas, memória, linguagem, atenção e mudanças comportamentais. Sintomas comportamentais, incluindo agitação, alucinações e apatia, foram proeminentes em DA-SD e demência prodrômica e podem servir como indicadores iniciais.

**Conclusão:**

A demência precoce em indivíduos com SD é caracterizada por declínios cognitivos e comportamentais, com sintomas comportamentais possivelmente atuando como marcadores diagnósticos precoces.

## INTRODUCTION

Life expectancy in individuals with Down syndrome (DS) has increased significantly in recent decades, resulting in a growing and aging population^1^ ^,^ ^2^. Studies show that the population with DS in the United States grew from about 50 thousand in 1950 to more than 200 thousand in 2010, with an aging trend due to increased survival^1^ ^,^ ^2^. This scenario brings new challenges, as people with DS present a higher prevalence of chronic comorbidities, such as cardiovascular and respiratory diseases, which translate into higher rates of hospitalizations and medical costs^3^ ^,^ ^4^. Furthermore, aging in this population is strongly associated with an increased risk of dementia, particularly Alzheimer’s disease (AD), which highlights the importance of appropriate cognitive assessments^5^.

Dementia in DS is most commonly associated with AD, with approximately 70% of individuals over the age of 50 displaying clinical signs of cognitive decline^5^. In comparison, the prevalence of AD in the general population over 65 years is about 10-15%, showing that trisomy 21 increases the risk multifold^6^ ^,^ ^7^. Beyond prevalence data, a recent systematic review highlighted that several interventions, including pharmacological, exercise, environmental, and cognitive training approaches, show small-to-moderate but significant effects in improving AD-related outcomes in individuals with DS, underscoring the urgent need for consistent and validated cognitive measures to better assess efficacy^8^. In parallel, large-scale initiatives such as the Investigation of Co-Occurring Conditions across the Lifespan to Understand Down Syndrome (NIH INCLUDE) project have expanded the research infrastructure and clinical trial capacity for DS, creating opportunities to implement and validate such outcome measures in a more systematic and representative manner^9^.

Cognitive decline in individuals with DS is increasingly recognized as a heterogeneous process that extends beyond memory deficits^10^. Executive dysfunction and behavioral symptoms frequently emerge during prodromal dementia (a transitional phase preceding the clinical diagnosis of dementia characterized by subtle cognitive, behavioral, or functional changes that do not yet meet full diagnostic criteria)^10^. These manifestations complicate diagnosis because they may be misinterpreted as personality changes or behavioral resistance rather than early indicators of neurodegeneration^11^. Similarly, mild cognitive impairment in DS refers to measurable deficits in one or more cognitive domains, such as memory, language, or executive function, that exceed age-related expectations but do not significantly impair daily functioning^12^. These prodromal and mild cognitive impairment phases may represent a continuum leading to early-stage AD, defined by mild but clinically evident cognitive and functional decline associated with pathologic changes characteristic of AD^13^.

Behavioral and neurological features (including apathy, agitation, seizures, motor dysfunction, and sleep disturbances) are also frequently observed in aging individuals with DS and may simulate features of traditional cognitive impairments^14^. This complex interplay underscores a diagnostic gap, since existing assessment tools are often adapted from general-population instruments, lack sensitivity to DS-specific manifestations, and may underdiagnose or misclassify dementia due to overlapping symptoms, floor effects, or caregiver-report bias^15^.

Given the exceptionally high prevalence of AD in this population and growing need for cognitive assessment tools specific for DS^8^, there is a pressing need for systematic evidence synthesis to clarify which cognitive and behavioral measures are most reliable for early detection in DS. Such synthesis can inform clinical practice by identifying tools that enable timely and accurate diagnosis, facilitate individualized care planning, and improve quality of life. It can also guide researchers by standardizing outcome measures, and prioritizing domains for future investigation.

In this manner, this study aimed to review and present the cognitive and behavioral characteristics of individuals with DS during the dementia process, along with the instruments used for cognitive evaluation in this population.

To address this gap, the guiding research question was structured according to the PCC acronym (P = Population; C = Concept; C = Context), which led to the following formulation: “How are cognitive and behavioral characteristics of dementia presented in cases within the context of aging in individuals with Down Syndrome?”.

## METHODS

### Protocol and reporting

This review followed the Preferred Reporting Items for Systematic Reviews and Meta-Analyses (PRISMA)^16^ guidelines and adhered to a protocol registered on Open Science Framework - OSF (https://doi.org/10.17605/OSF.IO/C39FY), to ensure transparency of methods and reproducibility. The protocol scope aims to set the bases for the present systematic review. The objective set is to examine cognitive changes and assessment tools in people with Down syndrome and dementia during aging, following PRISMA guidelines.

### Search strategy

The search strategy was developed using Medical Subject Headings (MeSH) and related keywords for Down syndrome and dementia-related cognitive and behavioral outcomes. The search string used was: (“Down Syndrome”) AND ((Dementia) OR (“Alzheimer Disease”) OR (“Frontotemporal Dementia”) OR (“Mixed Dementia”) OR (“Dementia, Vascular”) OR (“Lewy Body Disease”)) AND (Cognition) AND ((“Cognitive Dysfunction”) OR (“Cognitive Decline”) OR (“Cognitive Impairment”)) AND ((“Behavioral Symptoms”) OR (“Neurologic Manifestations”)).

The search was executed in August 2024 in the following electronic databases: United States National Library of Medicine (PubMed), Scopus, Web of Science, and Virtual Health Library (BVS). To prioritize recent evidence, we included studies published between 2014 and 2024 in English, Portuguese, or Spanish.

### Eligibility criteria

Inclusion criteria:

human studies addressing dementia in people with Down syndrome;
observational studies (cross-sectional, case-control, cohort, longitudinal, retrospective) and randomized controlled trials;
studies reporting data on screening and/or diagnosis of dementia in individuals with Down syndrome;
publications in English, Portuguese, or Spanish, from 2014-2024.

Exclusion criteria:

animal studies;
review articles;
articles in progress;
conference proceedings and abstracts.

### Study selection and data management

Retrieved records were imported into Rayyan software, as it was employed to organize and screen the retrieved records. This software facilitated blinded, independent review by multiple researchers and provided automated tools for identifying duplicates and resolving conflicts. Thereby, Rayyan software improved the efficiency and reliability of the screening process. Two reviewers independently screened titles and abstracts (A.C.L.N.; G.R.L.B.F.). Inter-rater reliability was documented using percent agreement between reviewers at each stage to ensure consistency of judgments. Agreement between reviewers was consistently high, with concordance ranging from approximately 80 to 90% across stages. Disagreements were resolved by a third reviewer (A.I.G.P.S.). Records selected at the title/abstract stage were exported to Excel for full-text reading. A final eligibility decision was made after a full-text review and the data were extracted into a structured spreadsheet.

### Data extraction

The following data were extracted from each included study: article title, year of publication, authors, study design, country, population characteristics (sample size and profile), age range, study objectives, methodology/design, instruments used for dementia diagnosis, other cognitive assessment instruments, main outcomes and conclusions, and information required for risk-of-bias assessment.

### Risk-of-bias assessment

The risk of bias was assessed using the Strengthening the Reporting of Observational Studies in Epidemiology (STROBE) checklist^17^. For this evaluation, the authors developed an original scoring system based on the STROBE criteria and the approach proposed by Olmos et al.^18^ Each of the 14 items was independently evaluated by two authors (A.C.L.N., E.P.P.N.) and classified on a scale from 0 to 2 points: 0 indicating non-compliance, 1 indicating partial compliance, and 2 indicating full compliance^18^. Agreement on risk of bias decision was very high, with concordance exceeding 90%. Discrepancies in scoring were resolved by a third evaluator (A.I.G.P.S.). The scores were systematically recorded in an Excel spreadsheet, which facilitated both data organization and the quantification of the risk of bias. The total possible score was 28 points, with the risk of bias categorized into three levels: low risk (22-28 points; 80-100%), moderate risk (14-21 points; 50-79%), and high risk (0-13 points; below 50%)^18^. Finally, the percentage of adequacy for each article was individually calculated, and classification was performed according to the categories mentioned above. This tool ensured a rigorous and detailed analysis of the methodological quality of the reviewed studies.

### Data synthesis

Due to the substantial heterogeneity among the included studies (particularly in study designs, diagnostic criteria, outcome measures, and statistical reporting), a quantitative synthesis (meta-analysis) was not feasible. Many studies did not report sufficient data (as means, standard deviations, or effect sizes) to permit pooling. Therefore, we conducted a narrative synthesis to summarize and compare findings across studies. Extracted data were synthesized narratively, focusing on the presentation of cognitive and behavioral characteristics of dementia in aging individuals with Down syndrome, and integrating study quality and heterogeneity in the interpretation of findings. Where appropriate, results were tabulated to display study characteristics, instruments used, and main outcomes.

## RESULTS

### Study selection

**Figure 1.**
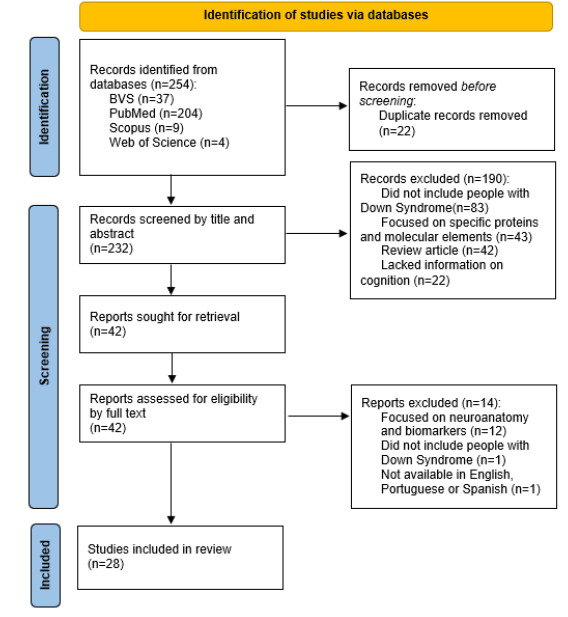
Preferred Reporting Items for Systematic Reviews and Meta-Analyses (PRISMA) 2020 flow diagram for new systematic reviews which included searches of databases and registers only.

The search identified 254 articles. After removing 22 duplicates, 232 studies were screened by title and abstract. Among these, 190 were excluded and 42 studies were retrieved for full-text review. In the final screening stage, 42 articles were evaluated in full text, of which 14 were excluded. Ultimately, 28^19^ ^,^ ^20^ ^,^ ^21^ ^,^ ^22^ ^,^ ^23^ ^,^ ^24^ ^,^ ^25^ ^,^ ^26^ ^,^ ^27^ ^,^ ^28^ ^,^ ^29^ ^,^ ^30^ ^,^ ^31^ ^,^ ^32^ ^,^ ^33^ ^,^ ^34^ ^,^ ^35^ ^,^ ^36^ ^,^ ^37^ ^,^ ^38^ ^,^ ^39^ ^,^ ^40^ ^,^ ^41^ ^,^ ^42^ ^,^ ^43^ ^,^ ^44^ ^,^ ^45^ ^,^ ^46^ articles were included in the review (Figure 1).

**Figure 2.**
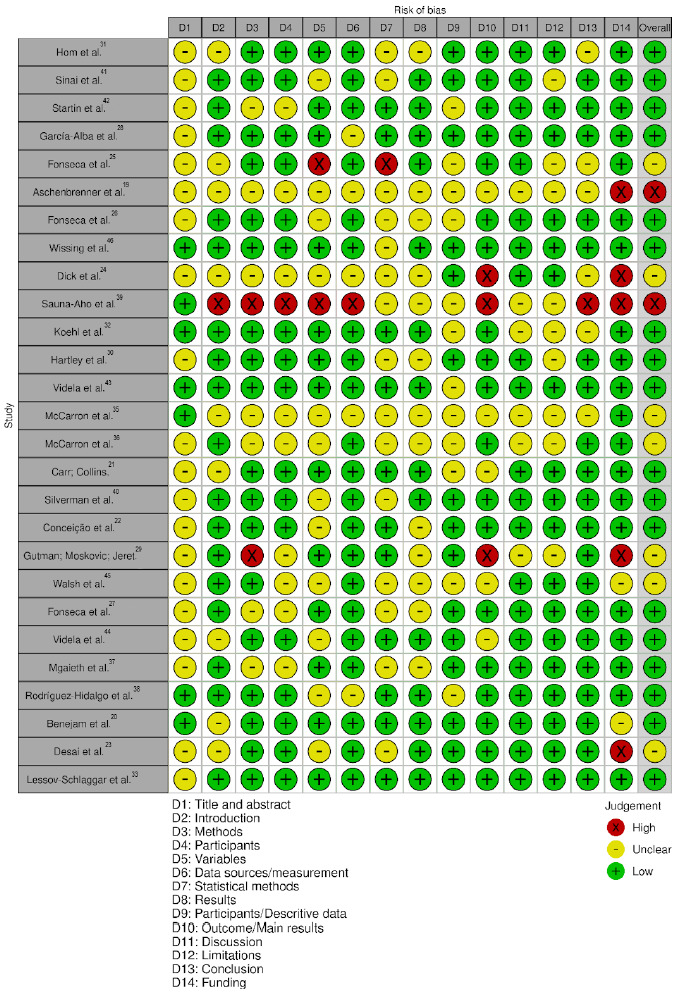
Quality assessment of studies using Strengthening the Reporting of Observational Studies in Epidemiology (STROBE) criteria to evaluate risk of bias.

Evaluation of the selected studies using the STROBE checklist with 14 items showed that the studies mostly had a low risk of bias. The quality assessment scores were presented in Supplementary Material Table S1 (available at https://www.demneuropsy.org/wp-content/uploads/2025/11/DN-2025.0308-Supplementary-Material.docx). Details on the quality assessment of the studies can be seen in Figure 2 ^19^ ^,^ ^20^ ^,^ ^21^ ^,^ ^22^ ^,^ ^23^ ^,^ ^24^ ^,^ ^25^ ^,^ ^26^ ^,^ ^27^ ^,^ ^28^ ^,^ ^29^ ^,^ ^30^ ^,^ ^31^ ^,^ ^32^ ^,^ ^33^ ^,^ ^35^ ^,^ ^36^ ^,^ ^37^ ^,^ ^38^ ^,^ ^39^ ^,^ ^40^ ^,^ ^41^ ^,^ ^42^ ^,^ ^43^ ^,^ ^44^ ^,^ ^45^ ^,^ ^46^.

The summary of the results from the articles collated, including details of the authors, year of publication, study design, population sample, assessment instruments, characteristics identified and risk of bias can also be found in Supplementary Material Table S1.

### Characteristics of the studies

Regarding the design of the studies included in the review, 16 studies^19^ ^,^ ^20^ ^,^ ^22^ ^,^ ^24^ ^,^ ^25^ ^,^ ^26^ ^,^ ^27^ ^,^ ^28^ ^,^ ^30^ ^,^ ^31^ ^,^ ^34^ ^,^ ^39^ ^,^ ^41^ ^,^ ^42^ ^,^ ^45^ ^,^ ^46^ (57%) were cross-sectional, ten^21^ ^,^ ^23^ ^,^ ^29^ ^,^ ^33^ ^,^ ^35^ ^,^ ^36^ ^,^ ^38^ ^,^ ^40^ ^,^ ^43^ ^,^ ^44^ (36%) were longitudinal, and two^32^ ^,^ ^37^ (7%) were both study types (Supplementary Material Table S1).

The median of the sample was 92, with the smallest study having 14^29^ participants, and the largest having 632^43^. Regarding the participants’ sex, male participants outnumbered female participants in the majority of the included studies^19^ ^,^ ^22^ ^,^ ^23^ ^,^ ^25^ ^,^ ^26^ ^,^ ^27^ ^,^ ^30^ ^,^ ^33^ ^,^ ^34^. The percentage of men in samples varied from 0 to 64%, with an average of 45.4% (standard deviation = 18.3%). This way, the percentage of women in samples varied from 36 to 100%, with an average of 55.5% (standard deviation = 18.3%).The diagnostic groups in the samples included people with stable cognition^22^ ^,^ ^23^ ^,^ ^25^ ^,^ ^26^ ^,^ ^27^ ^,^ ^28^ ^,^ ^30^ ^,^ ^31^ ^,^ ^34^ ^,^ ^38^ ^,^ ^41^ ^,^ ^43^ ^,^ ^44^, mild cognitive impairment^28^ ^,^ ^30^ ^,^ ^31^ ^,^ ^38^ or prodromal dementia^22^ ^,^ ^25^ ^,^ ^26^ ^,^ ^27^ ^,^ ^34^ ^,^ ^42^ ^,^ ^43^ ^,^ ^44^ and dementia^19^ ^,^ ^20^ ^,^ ^21^ ^,^ ^22^ ^,^ ^23^ ^,^ ^24^ ^,^ ^25^ ^,^ ^26^ ^,^ ^27^ ^,^ ^28^ ^,^ ^29^ ^,^ ^30^ ^,^ ^34^ ^,^ ^37^ ^,^ ^38^ ^,^ ^39^ ^,^ ^41^ ^,^ ^42^ ^,^ ^43^ ^,^ ^44^ ^,^ ^45^. In 44%^20^ ^,^ ^21^ ^,^ ^32^ ^,^ ^33^ ^,^ ^35^ ^,^ ^36^ ^,^ ^37^ ^,^ ^39^ ^,^ ^40^ ^,^ ^42^ ^,^ ^45^ ^,^ ^46^ of the studies, there was no or little information on the diagnostic group of the participants (Table 1).

**Table 1.** Characteristics of patients with Down syndrome in included studies.

Author	Age	Gender M (F)	Diagnostic group		
			Stable	MCI/PD	Dementia
Aschenbrenner et al.^19^	≥35	162 (150)	-	-	312
Benejam et al.^20^	≥ 18 years	-	-	-	AD: 15
Carr & Collins^21^	6 weeks to 50 years old.	-	-	-	5
Conceição et al.^22^	≥20	38 (28)	36	PD: 10	AD: 20
Desai et al.^23^	≥40	31 (21)	40	0	AD: 12
Dick et al.^24^	MA: 51.11 SD: 6.71	-	0	0	AD: 59
Fonseca et al.^25^	MA: 51.11 SD: 6.71	59 (33)	63	PD: 18	AD: 11
Fonseca et al.^26^	>30	93 (69)	113	PD: 24	AD: 24
Fonseca et al.^27^	30 to 64 years old. MA: 42.43 SD: 8.48	58 (34)	62	PD: 17	AD: 13
García-Alba et al.^28^	MA: 49.93 SD: 0.60	16 (25)	14	MCI: 14	AD: 13
Gutman et al.^29^	>40. MA: 52	4 (10)	0	0	AD: 14
Hartley et al.^30^	25 to 81 years old. MA: 45.19 SD = 9.88	199 (162)	263	MCI: 53	45
Hom et al.^31^	40 to 81 years old.	85 (59)	103	MCI: 41	0
Koehl et al.^32^	33 to 50 years old. MA: 41.31 SD: 10.57	32 (40)	41	7	13
Lessov-Schlaggar et al.^33^	18 to 55 years old. MA: 33.2 SD = 9.7	19 (15)	-	-	-
Mattar et al.^34^	MA: 42.43 SD: 8.48	58 (34)	62	PD:17	13
McCarron et al.^35^	≥ 35	0 (77)	-	-	-
McCarron et al.^36^	>35	0 (77)	-	-	-
Mgaieth et al.^37^	MA:49.59 SD: 7.95	150 (152)	-	-	37
Rodríguez-Hidalgo et al.^38^	MA: 46.65 SD: 5.08	46 (37)	48	MCI: 24	AD: 11
Sauna-Aho et al.^39^	> 36 MA: 50	34 (28)	-	-	AD: 14
Silverman et al.^40^	> 36	52 (133)	-	-	-
Sinai et al.^41^	45.1 to 64.9 years old. MA: 52.7 SD: 6.06	23 (26)	30	-	19
Startin et al.^42^	16 to 60 yearsold	149 (148)	-	PD: 44	45
Videla et al.^43^	MA: 40.9	290 (272)	416	PD: 62	84
Videla et al.^44^	MA: 40.9	340 (292)	436	PD: 69	AD: 127
Walsh et al.^45^	MA: 49.8 SD: 8.9	63 (51)	-	-	71
Wissing et al.^46^	21 to 83 years old. MA: 46	9 (91)	-	-	-

Abbreviations: M, male; F, female; MCI, mild cognitive impairment; PD, prodromal dementia; AD, Alzheimer’s disease; MA, mean age; SD, standard deviation.

Source: Authors, 2024.

Regarding the assessment instruments employed to evaluate participants’ cognitive function, the most frequently used were the Dementia Scale for Mentally Subnormal Elderly, cited in six studies^23^ ^,^ ^30^ ^,^ ^31^ ^,^ ^35^ ^,^ ^36^ ^,^ ^40^, with an expanded version referenced in one study^31^. The Dementia Questionnaire for People with Learning Disabilities (DLD) was also used in six studies^23^ ^,^ ^30^ ^,^ ^32^ ^,^ ^37^ ^,^ ^40^ ^,^ ^42^. The Cambridge Cognitive Examination for Older Adults with Down Syndrome (CAMCOG-DS) appeared in seven articles^19^ ^,^ ^25^ ^,^ ^27^ ^,^ ^28^ ^,^ ^34^ ^,^ ^43^ ^,^ ^44^, often in conjunction with the Cambridge Examination for Mental Disorders of Older People with Down’s Syndrome and Others with Intellectual Disabilities (CAMDEX-DS), except in one study^28^, where CAMDEX-DS was solely utilized as a diagnostic tool. The latter was also employed independently in two studies^22^ ^,^ ^26^. The modified version of the Cued Recall Test (mCRT) was frequently used as well, being mentioned in five articles^19^ ^,^ ^20^ ^,^ ^30^ ^,^ ^43^ ^,^ ^44^, like the Severe Impairment Battery, cited in four^19^ ^,^ ^24^ ^,^ ^32^ ^,^ ^45^. Further details can be found in Supplementary Material Table S1.

### Diagnosis and cognitive assessment

This review did not identify any instruments capable of diagnosing dementia in DS patients. Though early and accurate diagnosis is critical for managing dementia in DS^47^ ^,^ ^48^, one study^25^ highlighted that this process is particularly challenging in Brazil due to the lack of validated assessment tools for this population^25^. That study, by Fonseca et al., evaluated the validity and reliability of CAMDEX-DS for diagnosing dementia in adults with DS in Brazil. The diagnostic accuracy was 96.7%, with 100% sensitivity for AD and 88.9% for prodromal dementia, compared to the international criteria for dementia diagnosis as the reference standard^25^.

While CAMDEX-DS stood out as a validated option for dementia diagnosis in people with DS for the Brazilian population^25^, another tool has also shown promise in assessing cognitive, linguistic, and functional abilities in that group. The Arizona Battery for Communication Disorders of Dementia has been translated and culturally adapted for Brazil, having shown to be useful for assessing linguistic skills in adults and elderly people with DS^49^. That instrument has presented a strong correlation with functionality as measured by the Lawton Instrumental Activities of Daily Living scale^50^, Pfeffer Functional Activities Questionnaire^51^ and Katz Index^52^ scales^49^. However, it is important to mention that, while these three tools have been validated in Portuguese^53^ ^,^ ^54^, they were not specifically developed for individuals with DS^50^ ^,^ ^51^ ^,^ ^52^.

One study in the review assessed the validity of the Arizona Cognitive Test Battery (ACTB) in older adults with DS and evaluated its ability to distinguish between those with and without dementia^41^. The tool was able to identify differences in cognitive functions between groups with and without dementia, but scores on a number of ACTB tests were similar between the two groups^41^. Another study^55^ from Sinai, conducted in 2014, had already evaluated the ACTB in older adults with DS, reaching a similar conclusion^55^. It found that, while the ACTB could assess cognitive function, it lacked the sensitivity to differentiate between individuals with early-stage dementia and those without cognitive impairment^55^. The more recent study corroborates these findings, reinforcing that, although the ACTB can be a useful cognitive assessment tool, it does not reliably diagnose dementia in this population^41^ ^,^ ^55^.

In comparison with Severe Impairment Battery (SIB) and Box Praxis Test (BPT), DLD was more sensitive to early changes of incipient dementia, which indicated behavioral changes (delusions, outbursts of anger, nocturnal confusion, agitation and visual hallucinations) as early symptoms of dementia in DS^32^. CDR-QDS and CDR-IDS were effective in identifying cases of dementia and cognitive deficits in DS^33^.

Cognitive assessment in this study was organized around three core domains: memory (including short-term/working memory and long-term/episodic memory), executive function (processes such as working memory/updating, inhibition, cognitive flexibility/set-shifting, planning and monitoring), and language (receptive vs. expressive skills, phonology, vocabulary, syntax and pragmatic/useful communication). Defining these constructs makes it possible to interpret results consistently and to place findings in the broader DS literature concerning cognitive decline, where a characteristic cognitive profile is repeatedly reported^14^.

Memory appears in five studies as one of the earliest and most sensitive indicators of cognitive decline in individuals with DS^19^ ^,^ ^25^ ^,^ ^26^ ^,^ ^31^ ^,^ ^42^. Other investigations in scientific literature have further confirmed that hypothesis. For instance, Firth et al.^56^ reported that deficits in memory occurred in the beginning of the dementia process in progression to AD^56^. In addition, it has been found that declines in verbal and working memory not only serve as early indicators of dementia progression but also predict subsequent deterioration in other cognitive domains^57^. Regarding memory decline as an early or sensitive marker, three studies explicitly framed memory as an early/sensitive sign of decline^19^ ^,^ ^26^ ^,^ ^42^. Several other papers reported prominent memory impairment in AD-DS or age-related memory worsening^20^ ^,^ ^25^ ^,^ ^28^ ^,^ ^40^, but did not specifically label memory as an early marker in the way the three studies above did.

Benejam et al.^20^ highlighted that age-related memory deterioration is present in DS, even in those without dementia. In AD, however, memory decline is more severe, with progressive loss of episodic and semantic memory, a pattern consistent with Fonseca et al.^25^, who observed significantly worse memory performance in individuals with AD and DS (AD-DS) than in DS without dementia. Similarly, Krinsky-McHale et al.^58^ had already found that long-term storage and retrieval abilities declined sharply in individuals with early-stage AD, often preceding other symptoms by over a year^58^. This indicates that, while cognitive decline is a natural aspect of aging in DS, the memory deficits associated with AD-DS might be more pronounced and progressive.

Impairments in executive function are also critical in understanding the cognitive profile in DS. Fonseca et al.^26^ noted that, in prodromal dementia and DS, memory deficits are frequently accompanied by executive dysfunction, suggesting an intrinsic relationship between these domains^26^. Also, Startin et al.^42^ stated that concurrent deficits in memory and executive function may signal a more global cognitive decline^42^. Those observations are in accordance to Lautarescu et al.^13^, who have pointed to executive dysfunction as common in the pre-clinical and early stages of Alzheimer’s disease in people with Down syndrome.

Language deficits, although less frequently isolated in the reviewed studies, tend to parallel the deterioration observed in memory and executive function. Fonseca et al.^25^ indicated that, as DS individuals progress to more advanced stages (prodromal dementia and AD), impairments in language become increasingly evident. This pattern is corroborated by external literature; for instance, Pulsifer et al.^59^ reported that early changes in language comprehension and expression often accompany memory decline, affecting both communication and social interaction as the disease advances. Similarly, Pulsifer et al.^59^ also found that language deterioration is not only a common feature of cognitive decline in DS but also serves as a significant predictor of dementia onset. This way, progressive deficits in language impact social engagement and quality of life, while they may reflect ongoing cognitive decline.

### Cognitive changes over time

The progression of early cognitive decline in episodic memory, visuospatial skills, and general dementia symptoms showed similarities across all premorbid intellectual disability DS groups in two studies^30^ ^,^ ^43^. In other words, the idea is that, regardless of whether an individual with DS has mild, moderate, or severe intellectual disability to begin with, AD will progress at a similar pace, as was also suggested by Shimizu et al^60^. Furthermore, two studies showed a strong association between high risk of progressive cognitive decline in DS and the onset of symptomatic AD^36^ ^,^ ^43^, which has been supported by evidence linking age and cognitive impairment in this population^61^. Considerably, this aligns with other evidence suggesting that AD is currently the main medical concern and leading cause of death among individuals with DS^48^.

In one study^30^, the average age of diagnosis of mild cognitive impairment in DS and AD-DS also did not differ by premorbid intellectual disability level, occurring in the early to mid-50 s, respectively^30^, which is consistent with recent evidence: subtle memory/executive deficits can appear from 35 years of age, prodromal AD appears with a mean age of presentation of 50.8 years, while dementia presents with a mean age of onset of 53.8 years of age^48^. Some assessment tools in one of the included studies^35^ also showed an average diagnosis age of 55.41 years (standard deviation=7.14) and a median survival of seven years post-diagnosis^35^. Those tools were the DLD, the Severe Impairment Battery and the Brief Praxis Test^35^.

Tools such as CAMCOG-DS have proven effective in identifying cognitive deficits at different stages of the disease in eight studies^19^ ^,^ ^25^ ^,^ ^27^ ^,^ ^28^ ^,^ ^34^ ^,^ ^43^ ^,^ ^44^. Along with the mCRT^19^ ^,^ ^20^ ^,^ ^30^ ^,^ ^43^ ^,^ ^44^, this scale has demonstrated high accuracy in detecting early stages of DA in DS, reinforcing their clinical use as indicators for more in-depth evaluations^62^.

### Behavioral assessment

One study found that, when comparing AD-DS with both the group of individuals with prodromal dementia and DS and the group of cognitively stable individuals with DS, AD-DS had a higher prevalence (frequency) of hallucination, agitation, apathy, and nighttime behavior disturbances^27^. Compared with people with DS that had stable cognition, both the group of individuals with DS and prodromal dementia and the group of AD-DS had a higher prevalence of aberrant motor behavior^27^. Additionally, another study found that signs indicating dementia in people with DS included forgetfulness, loss of energy, reduced self-care skills and weight change^39^.

One study revealed that, in comparison with individuals with severe/profound intellectual disability without dementia, those with dementia had a decline in activities of daily living, behavioral and psychological symptoms like increased irritable, anxious, apathetic behavior and decreased eating/drinking^46^. On this topic, Dekker et al.^63^, optimized and validated the Behavioral and Psychological Symptoms of Dementia - DS scale to identify behavioral changes and early signals of dementia in individuals with DS^63^. This advancement has been fundamental in broadening the conversation around the role of behavioral symptoms in dementia diagnosis and indicating the possibility of considering behavioral shifts as potential early markers of the disease^63^.

In that context, DLD has its use encouraged by recent evidence, due to the high levels of agreement between its scores and clinicians’ diagnoses, as well as its good sensitivity and specificity, being effective in identifying deterioration in cognitive and social skills in adults with DS over time^64^. Corroborating this, one study found that DLD was more sensitive than the Severe Impairment Battery and the Brief Praxis Test in detecting early changes of incipient dementia, such as delusions, outbursts of anger, nocturnal confusion, agitation and visual hallucinations as early symptoms of dementia in DS^32^. Notably, this might also mean that behavioral changes could also serve as indicators of cognitive decline. This highlights the need for further studies to assess whether behavioral and neuropsychiatric symptoms can be considered early markers of dementia in DS. Future research should explore the effectiveness of assessment tools specifically designed to evaluate behavioral symptoms, investigating their potential role in detecting early-stage dementia and understanding how these symptoms relate to cognitive decline over time.

## DISCUSSION

### Limitations of the studies included

It is important to highlight some limitations of the studies included in this systematic review, such as the ‘small sample size,’ classified based on the studies’ own limitation sections^21^ ^,^ ^22^ ^,^ ^25^ ^,^ ^27^ ^,^ ^28^ ^,^ ^29^ ^,^ ^30^ ^,^ ^32^ ^,^ ^33^ ^,^ ^35^ ^,^ ^36^ ^,^ ^42^ ^,^ ^45^, particularly when they stated that their sample size influenced certain effect measures. This limitation can lead to reduced statistical power and limited generalizability, making it challenging to draw definitive conclusions about the broader population.

Furthermore, it was observed that only two out of the 27 analyzed studies^24^ ^,^ ^30^ considered race and ethnicity. One limitation of this is the difficulty in distinguishing race from ethnicity, which could hinder racial and ethnic differences from the samples.

Data provided by caregivers limited the sensitivity of the data obtained in the studies, which was a limitation in some articles^21^ ^,^ ^23^ ^,^ ^27^. For example, in one article^27^, neuropsychiatric symptoms were identified through caregiver reports, which may be biased compared to direct observation of participant behavior. Additionally, family members or even healthcare professionals may have reported symptoms of other conditions that mimic dementia^46^, compromising the sensitivity of the sample. This limitation could affect the sensitivity of detecting symptoms, potentially leading to an underestimation or misclassification of dementia severity. These limitations can restrict the generalizability of the results to the general population. Therefore, this was one of the limitations in conducting the current review. Additional studies on a larger and more precise scale are needed.

Future research should recruit larger, more representative samples to strengthen statistical power and generalizability. Studies should also systematically include race and ethnicity, with clear definitions, to capture potential differences across groups. To reduce bias, informant reports should be complemented with direct assessments, while longitudinal designs and standardized assessment protocols might improve sensitivity to change and facilitate comparisons across studies.

### Limitations of this review

This systematic review is subject to limitations that warrant consideration. Firstly, the quality of included studies varied significantly. A STROBE assessment revealed that 7.14%^19^ ^,^ ^39^ of the 28 included studies exhibited a high risk of bias, while 25%^23^ ^,^ ^24^ ^,^ ^25^ ^,^ ^29^ ^,^ ^35^ ^,^ ^36^ ^,^ ^45^ demonstrated a moderate one. This leaves only 67.85%^20^ ^,^ ^21^ ^,^ ^22^ ^,^ ^26^ ^,^ ^27^ ^,^ ^28^ ^,^ ^30^ ^,^ ^31^ ^,^ ^32^ ^,^ ^33^ ^,^ ^34^ ^,^ ^37^ ^,^ ^38^ ^,^ ^40^ ^,^ ^41^ ^,^ ^42^ ^,^ ^43^ ^,^ ^44^ ^,^ ^46^ of the studies with a low risk of bias, potentially impacting the robustness of the findings. The risk of bias assessment showed that, overall, the included studies presented a low risk across most domains, demonstrating good methodological quality and clear reporting of methods, results, discussion, and conclusion. However, some critical issues were identified. The main concerns were observed in D1 (Title and Abstract), D7 (Statistical Methods), D10 (Outcome/Main Results), and D14 (Foundation), where several studies showed either unclear or high risk, suggesting shortcomings in transparency, reporting accuracy, and methodological rigor (Figure 2).

Secondly, the language restriction to English, Portuguese, and Spanish may have resulted in the exclusion of relevant studies published in other languages.

Finally, the heterogeneity of the included studies in terms of participant populations and cognitive assessment instruments could have influenced the overall results. This variability may have hindered the ability to draw definitive conclusions due to the difficulty in comparing findings across diverse study designs.

### Practical implications

This review highlights memory, executive function, and behavioral symptoms as potential early indicators of dementia in individuals with DS, underscoring the value of adapted tools such as the CAMCOG-DS and mCRT for clinical use. For clinicians, incorporating these tools into routine assessments can support earlier and more accurate diagnosis, allowing care plans to be tailored to the DS cognitive profile. For caregivers, awareness of early signs can prompt earlier evaluation, mirroring strategies already promoted in the broader AD population. For policymakers, the findings reinforce the need for guidelines and training that standardize dementia screening in DS and ensure access to appropriate diagnostic resources. Future research should test whether early detection in DS, as shown in typical AD populations, leads to more effective interventions and improved quality of life. Strengthening collaboration between DS research and mainstream dementia research will be essential for translating early identification into meaningful clinical and social outcomes.

## DATA AVAILABILITY STATEMENT

The datasets generated and/or analyzed during the current study are available from the corresponding author upon reasonable request.
